# Factors associated with attention deficit/hyperactivity disorder among US children: Results from a national survey

**DOI:** 10.1186/1471-2431-12-50

**Published:** 2012-05-14

**Authors:** Ravi K Lingineni, Swati Biswas, Naveed Ahmad, Bradford E Jackson, Sejong Bae, Karan P Singh

**Affiliations:** 1 Department of Biostatistics, School of Public Health, University of North Texas Health Science Center, Fort Worth, TX, USA; 2 Department of Mathematics, University of Texas at Dallas, Richardson, TX, USA; 3 Department of Pediatrics, University of Mississippi Medical Center, Jackson, MS, USA; 4 Division of Preventive Medicine, Department of Medicine, University of Alabama at Birmingham, Birmingham, AL, USA; 5 Department of Biostatistics, School of Public Health, University of North Texas Health Science, Center, 3500 Camp Bowie Blvd, Fort Worth, TX, 76107, USA

**Keywords:** National Survey of Children’s Health, Neurobehavioral disorder, Obesity, Depression, Medication, TV usage, Participation in sports, Smoking status

## Abstract

**Background:**

The purpose of this study was to investigate the association between Attention Deficit/Hyperactivity Disorder (ADHD) and various factors using a representative sample of US children in a comprehensive manner. This includes variables that have not been previously studied such as watching TV/playing video games, computer usage, family member’s smoking, and participation in sports.

**Methods:**

This was a cross-sectional study of 68,634 children, 5–17 years old, from the National Survey of Children’s Health (NSCH, 2007–2008). We performed bivariate and multivariate logistic regression analyses with ADHD classification as the response variable and the following explanatory variables: sex, race, depression, anxiety, body mass index, healthcare coverage, family structure, socio-economic status, family members’ smoking status, education, computer usage, watching television (TV)/playing video games, participation in sports, and participation in clubs/organizations.

**Results:**

Approximately 10% of the sample was classified as having ADHD. We found depression, anxiety, healthcare coverage, and male sex of child to have increased odds of being diagnosed with ADHD. One of the salient features of this study was observing a significant association between ADHD and variables such as TV usage, participation in sports, two-parent family structure, and family members’ smoking status. Obesity was not found to be significantly associated with ADHD, contrary to some previous studies.

**Conclusions:**

The current study uncovered several factors associated with ADHD at the national level, including some that have not been studied earlier in such a setting. However, we caution that due to the cross-sectional and observational nature of the data, a cause and effect relationship between ADHD and the associated factors can not be deduced from this study. Future research on ADHD should take into consideration these factors, preferably through a longitudinal study design.

## Background

The diagnosis of psychiatric, behavioral, and learning disorders has increased over the past decade. Attention Deficit/Hyperactivity Disorder (ADHD) is one of the most common childhood neurobehavioral disorders in the U.S [1,2]. According to the Diagnostic and Statistical Manual of Mental Disorders (DSM-IV), ADHD is characterized by pervasive and developmentally inappropriate symptoms such as severe lack of attention, impulsive behavior, and hyperactivity that affects children and persists through adulthood in 30–50% of ADHD affected children [3-6]. It is a multi-factorial and clinically heterogeneous disorder that affects about 9% to 15% of school going children in the US [7-10]. Previous studies, as of 2007, have found that the percentage of parent reported (ever) ADHD diagnosed children below 17 years of age was 9.5% or about 5.4 million, which represents a 22% increase in four years from 2003 to 2007 [11-14].

In the past 15 years, studies on uncovering the etiology of ADHD focused mainly on the association between ADHD and genetic factors, specifically DRD2 and DRD4 genes [15,16]. Some other studies have shown an association of ADHD with factors such as obesity, depression, anxiety, sex, age, race, asthma, cigarette smoking, family structure, and socio economic status (SES) [17-27]. Many of these studies showed a significant association between ADHD and body mass index (BMI), however, these studies did not consider factors that may be common for both ADHD and BMI such as participation in sports, clubs, computer usage, and watching television/playing video games [27-30]. Some studies have shown that having ADHD increases the chance of early initiation of smoking habit in children [26,27]. Also, the effect of mother’s smoking during pregnancy on ADHD has been previously studied [31,32]. However, the effect of family members’ smoking on children’s ADHD has not been considered. Thus, there is a need for a more comprehensive study of factors. Our study considers the potential factors from previous studies as well as the factors not considered earlier together. The purpose of this study was to explore the association between ADHD diagnosis and many factors together using a nationally representative sample, in particular, the National Survey of Child Health (NCSH) of the US. Even though our study cannot establish any of these factors as risk/protective factors due to the cross-sectional and observational nature of the data, we believe this type of comprehensive study on association is lacking in the ADHD literature and this article aims to fill this gap.

## Methods

### NSCH data

NSCH is a population-based, cross-sectional, random-digital-dialing survey using a complex, multi-cluster, probability sampling design [33]. This survey was sponsored by the Department of Health and Human Services (DHHS), Maternal and Child Health Bureau in partnership with the National Center for Health Statistics, which is a part of the Center for Disease Control and Prevention [34]. The data were collected by random-digital-dialing households with children under 18 years of age from each of the 50 states and the District of Columbia during the period of April 2007 to July 2008. A child was randomly selected from each of the sampled households to be the subject of the survey. The parent or guardian who knew the most about the selected child’s health and health care was interviewed with questions over the telephone. The interview was conducted in both English and Spanish, and consisted of questions regarding demographics, child’s health and functional status, health insurance coverage, health care access and utilization, medical home, family functioning, parental health, and neighborhood/community characteristics. A total of 91,642 children of age 17 years or under, with an overall weighted response rate of 55.3% were included in NSCH 2007 study. Population based estimates were obtained by assigning sampling weights to each sampled child for whom an interview was completed. Detailed information about the design of the NSCH is available at http://www.nschdata.org.

### Study sample

According to the DSM-IV, the diagnosis of ADHD requires the child to experience ADHD characteristics such as lack of attention, impulsive behavior, and hyperactivity in at least two different settings, namely home and school; while previous studies also show that the earliest onset age for a child to be diagnosed and treated for ADHD can be below 7 years [35]. As one of the two settings required for the diagnosis of ADHD is school, we considered children between 5 to 17 years old to be our study sample. There were a total of 68,634 responses given by the parents or guardians of children aged 5 to 17 years. Since the NSCH is a population based survey, the selected sample is a representative sample of children aged 5 to 17 years in the US.

### Variables

The primary dependent variable used in our study was ascertained through the following question to the parent or guardian: “Has a doctor or health professional ever told you that selected child (S.C.) has attention deficit disorder or attention deficit hyperactive disorder, that is, ADD or ADHD?”. We categorized the variable into: ‘ADHD’ if the response was ‘Yes’, and ‘No ADHD’ if the response was ‘No’. The psychological factors included in the study were depression (DEP; “Has a doctor or other health care provider ever told you that S.C. had depression?”) and anxiety (ANX; “Has a doctor or other health care provider ever told you that S.C. had anxiety problems?’). The NSCH has a sex- and age-specific derived BMI variable that is categorized into underweight (<5^th^ percentile), normal weight (≥5^th^ and <85^th^ percentile), at risk of overweight (≥85^th^ and <95^th^ percentile) and overweight (≥95^th^ percentile). To explicate, we refer to the last two categories as overweight and obese, respectively. Other independent variables included in the analysis were sex, race/ethnicity (Non-Hispanic White vs. Non- Hispanic Black, Hispanic, Other), highest level of education (EDUC) attained by anyone in the household (More than high school vs. High school graduate or less), family structure (FAMILY; Two parent including biological, step, or adopted vs. Other including single mother), dichotomized poverty level based on DHHS Federal Poverty guidelines (POVERTY; ≤ 200% vs. > 200%), healthcare coverage (INS; Having any health insurance such as Health Maintenance Organizations (HMOs) or Medicaid vs. Not having healthcare coverage), participation in sports (SPORTS; S.C. on a sports team or taking sports lessons after the school or on weekends vs. Not participating in sports), participation in clubs (CLUBS; S.C. on any clubs or organizations after school or on weekend vs. Not participating in clubs), daily average computer usage (COMP) for purposes other than school work (≥ 1 hour vs. < 1 hour), daily average television watching/playing video games (TV; ≥ 1 hour vs. < 1 hour), family member’s smoking status (SMOKE; Cigarettes, cigars, or pipe tobacco used by anyone in the household vs. No one in the household smokes) [17-32].

We also considered the effect of medication on the association between ADHD and BMI, as most of the medications prescribed for ADHD may have anorectic effects [36]. To do this we utilized the question: “Is S.C. currently taking medication for ADD or ADHD?”. We combined responses to this question with the ones to the ADHD question mentioned earlier to create a variable with three categories: ‘ADHD and currently taking medication’ (ADHD-CM), ‘ADHD and not currently taking medication’ (ADHD-NCM), and ‘No ADHD’; ‘No ADHD’ was considered as the reference group. This trichotomized variable is used as a dependent variable in one of the models. We chose not to use this variable as our primary dependent variable due to several limitations with the medication use question and its discrepancy in sample size with the ADHD question (elaborated later in the Discussion section).

### Statistical analysis

We computed descriptive statistics based on unweighted sample sizes and weighted percentages for children aged 5 to 17 years. Next, we used chi-square tests for testing the association of each variable with ADHD. Then we performed logistic regression analysis with ADHD as the response variable and the independent variables specified above, first with each of the variables individually (bivariate analysis) and then with all of them in the model (multivariate analysis) to obtain unadjusted and adjusted odds ratios (OR), respectively. For the trichotomized dependent variable that incorporates the medication effect, we used the multinomial logistic regression with independent variables same as in our previous model. All analyses were carried out in SAS version 9.2 to account for the complex survey design of the study [37,38]. In particular, we performed domain analysis for the 5–17 years age group [39].

## Results

The descriptive statistics (Table 1) show that the prevalence of ADHD is about 10% (n = 7,137). In this study, 51.13% were male; 56.75% were non-Hispanic White, 14.93% were non- Hispanic Black and 19.77% were Hispanic. About 16.37% of the study subjects were obese while 63.19% were normal weight, and only 5.16% were underweight. DEP and ANX were reported in 4.50% and 5.31% of the children, respectively. Further, 62.31% were above the 200% POVERTY level specified by DHHS and around two thirds (66.35%) of the households had more than high school EDUC. Most of the children (90.43%) had INS, were (73.19%) living in two-parent FAMILY, had (73.62%) no one SMOKE in the household. Additionally, 58.30% and 56.89% were in SPORTS and CLUBS, respectively; and 61.36% and 83.99% had used COMP for other than schoolwork and watched TV on an average for ≥1 hour during a weekday, respectively. Finally, of the children classified as with ADHD, 66.72% take medication.

**Table 1:** Characteristics of subjects 5–17 years old, NSCH 2007

Variable	N (Unweighted)	Weighted % ± SE
ADHD classification		
Yes	7137	10.08 ± 0.28
No	61378	89.91 ± 0.28
BMI		
Underweight	2186	5.16 ± 0.27
Normal	29121	63.19 ± 0.60
Overweight	6754	15.26 ± 0.44
Obese	6040	16.37 ± 0.48
Sex		
Male	35677	51.13 ± 0.48
Female	32863	48.86 ± 0.48
Age: Median(IQR)		10.63 (6.51)
Depression		
Yes	3088	4.50 ± 0.21
No	65481	95.49 ± 0.21
Anxiety		
Yes	4125	5.31 ± 0.21
No	64428	94.68 ± 0.21
Race/Ethnicity		
Non- Hispanic White	46739	56.75 ± 0.51
Non-Hispanic Black	6908	14.93 ± 0.33
Hispanic	8006	19.77 ± 0.50
Other	5894	8.53 ± 0.29
Poverty		
≤200%	17008	37.68 ± 0.50
>200%	45873	62.31 ± 0.50
Family member’s Smoking status		
By any one in household	17221	26.37 ± 0.42
No one Smoke	50901	73.62 ± 0.42
Highest level of Education in the household		
Less than/High School Education	15624	33.16 ± 0.49
More than High School Education	52204	66.83 ± 0.49
Family structure		
Two parent -biological/step/adopted	51682	73.19 ± 0.43
Other - single mother/father/other	16532	26.80 ± 0.43
Healthcare coverage		
Yes	63154	90.43 ± 0.31
No	5343	9.56 ± 0.31
Participation in Sports		
Yes	40673	58.30 ± 0.50
No	23354	41.69 ± 0.50
Participation in Clubs		
Yes	40352	56.89 ± 0.51
No	23649	43.10 ± 0.51
Average computer usage during a weekday		
≥1 hour	30744	61.36 ± 0.54
<1 hour	19811	38.64 ± 0.54
Average TV usage during weekday		
≥1 hour	50080	83.99 ± 0.39
<1 hour	9898	16.01 ± 0.39
Current Medication Use (in ADHD group)		
Yes	3735	66.72 ± 1.65
No	1690	33.28 ± 1.65

Unweighted N = 68634.

SE: Standard Error; IQR: Inter-Quartile Range.

We summarize various factors by ADHD classification and report the corresponding p- values in Table 2. All the factors were statistically significant at the 0.05 level. In particular, children in the ADHD and No ADHD groups differ strikingly in many characteristics including: 70.90% vs. 48.90% males, 22.93% vs. 2.43% DEP, 23.73% vs. 3.22% having ANX, 39.60% vs. 24.86% having someone SMOKE, 59.76% vs. 74.74% living in a two-parent FAMILY, and 48.51% vs. 59.50% in SPORTS, respectively. The ORs and their confidence intervals (CI) from the bivariate analysis are shown in Table 3. The following variables showed significantly increased odds of being classified as having ADHD: when a child was male (OR 2.55, 95% CI 2.22–2.92); obese (OR 1.45, 95% CI 1.19–1.77); had DEP (OR 11.94, 95% CI 9.75–14.61); had ANX (OR 9.35, 95% CI 7.82–11.18); belonged to ≤ 200% POVERTY level (OR 1.33, 95% CI 1.17–1.51); had INS (OR 1.54, 95% CI 1.16–2.04); had someone SMOKE (OR 1.98, 95% CI 1.75–2.25); used COMP for ≥1 hour for the purpose other than school work in a weekday (OR 1.52, 95% CI 1.31–1.77); or watched TV for ≥1 hour (OR 1.82, 95% CI 1.55–2.13); than the respective reference group. Additionally, the odds of a child being diagnosed with ADHD increased 10% (OR 1.10, 95% CI 1.08–1.11) with every one year increase in age. A child was significantly less likely to be classified as having ADHD if he/she was living in a two-parent FAMILY (OR 0.50, 95% CI 0.44–0.57); was Hispanic (OR 0.51, 95% CI 0.40–0.65); had at least one of the parent/guardian with more than high school EDUC (OR 0.78, 95% 0.68–0.89); was in SPORTS (OR 0.64, 95% CI 0.57–0.73); or was in CLUBS (OR 0.78, 95% CI 0.69–0.88).

**Table 2:** Factors stratified by ADHD classification for subjects 5–17 years old, NSCH 2007

Variable	ADHD		NO ADHD		
	N	Weighted	N	Weighted	P-value
	**(Unweighted)**	**% ± SE**	**(Unweighted)**	**% ± SE**	
BMI					
Underweight	292	4.26 ± 0.58	1892	5.28 ± 0.36	<0.001
Normal	3418	58.57 ± 1.73	25664	63.84 ± 0.64	
Overweight	867	16.21 ± 1.34	5876	15.13 ± 0.46	
Obese	952	20.94 ± 1.48	5078	15.73 ± 0.51	
Sex					
Male	5068	70.90 ± 1.37	30536	48.90 ± 0.51	<0.001
Female	2063	29.09 ± 1.37	30754	51.09 ± 0.51	
Age: Median(IQR)	13 (5)	11.98 (5.69)^*^	12 (7)	10.46 (6.59)^*^	<0.001
Depression					
Yes	1505	22.93 ± 1.39	1573	2.43 ± 0.15	<0.001
No	5611	77.07 ± 1.39	59778	87.75 ± 0.15	
Anxiety					
Yes	1854	23.73 ± 1.21	2257	3.22 ± 0.19	<0.001
No	5260	76.26 ± 1.21	59084	96.77 ± 0.19	
Race/Ethnicity					
Non- Hispanic White	5110	63.62 ± 1.47	41567	56.00 ± 0.54	<0.001
Non-Hispanic Black	741	16.25 ± 1.06	6148	14.76 ± 0.35	
Hispanic	613	11.97 ± 1.22	7374	20.64 ± 0.54	
Other	586	8.14 ± 0.77	5292	8.58 ± 0.31	
Poverty					
≤200%	2205	43.73 ± 1.52	14759	36.94 ± 0.54	<0.001
>200%	4446	56.26 ± 1.52	41376	63.05 ± 0.54	
Family members’ Smoking status					
By any one in household	2453	39.60 ± 1.45	14724	24.86 ± 0.43	<0.001
No one Smoke	4645	60.39 ± 1.45	46182	75.13 ± 0.43	
Highest level of Education in the household					
Less than/High School Education	1938	38.31 ± 1.47	13641	32.54 ± 0.52	<0.001
More than High School Education	5137	61.68 ± 1.47	46993	67.45 ± 0.52	
Family structure					
Two parent -biological/step/adopted	4570	59.76 ± 1.45	47043	74.74 ± 0.44	<0.001
Other - single mother/father/other	2528	40.23 ± 1.45	13955	25.25 ± 0.44	
Healthcare coverage					
Yes	6719	93.35 ± 0.86	56333	90.12 ± 0.33	0.002
No	408	6.66 ± 0.86	4919	9.87 ± 0.33	
Participation in Sports					
Yes	3688	48.51 ± 1.47	36933	59.50 ± 0.54	<0.001
No	3333	51.48 ± 1.47	19969	40.49 ± 0.54	
Participation in Clubs					
Yes	3839	51.38 ± 1.48	36463	57.57 ± 0.54	<0.001
No	3179	48.61 ± 1.48	20417	42.42 ± 0.54	
Average computer usage during a weekday					
≥1 hour	3633	69.87 ± 1.52	27066	60.37 ± 0.58	<0.001
<1 hour	1677	30.12 ± 1.52	18115	39.62 ± 0.58	
Average TV usage during weekday					
≥1 hour	5788	90.02 ± 0.68	44204	83.24 ± 0.43	<0.001
<1 hour	847	9.97 ± 0.68	9041	16.75 ± 0.43	

Unweighted N = 68634.

SE: Standard Error; IQR: Inter-Quartile Range.

* Weighted Mean (Weighted SE).

**Table 3:** Unadjusted odds ratios for factors associated with ADHD classification, NSCH 2007

Variable	ADHD		
	OR	LCL	UCL
BMI			
Underweight	0.88	0.65	1.20
Normal	Ref	Ref	Ref
Overweight	1.17	0.95	1.44
Obese	1.45*	1.19	1.77
Sex			
Male	2.55*	2.22	2.92
Female	Ref	Ref	Ref
Age	1.10*	1.08	1.11
Depression			
Yes	11.94*	9.75	14.61
No	Ref	Ref	Ref
Anxiety			
Yes	9.35*	7.82	11.18
No	Ref	Ref	Ref
Race/Ethnicity			
Non- Hispanic White	Ref	Ref	Ref
Non-Hispanic Black	0.97	0.82	1.14
Hispanic	0.51*	0.40	0.65
Other	0.83	0.67	1.04
Poverty			
≤200%	1.33*	1.17	1.51
>200%	Ref	Ref	Ref
Family members’ smoking status			
At least one member smokes	1.98*	1.75	2.25
No one smokes	Ref	Ref	Ref
Highest level of Education in the household			
Less than/High School Education	Ref	Ref	Ref
More than High School Education	0.78*	0.68	0.89
Family structure			
Two parent -biological/step/adopted	0.50*	0.44	0.57
Other - single mother/father/other	Ref	Ref	Ref
Healthcare coverage			
Yes	1.54*	1.16	2.04
No	Ref	Ref	Ref
Participation in Sports			
Yes	0.64*	0.57	0.73
No	Ref	Ref	Ref
Participation in Clubs			
Yes	0.78*	0.69	0.88
No	Ref	Ref	Ref
Average computer usage during a weekday			
≥1 hour	1.52*	1.31	1.77
<1 hour	Ref	Ref	Ref
Average TV usage during weekday			
≥1 hour	1.82*	1.55	2.13
<1 hour	Ref	Ref	Ref

* Significant association at 0.05 level.

OR: Odds Ratio; LCL: 95% Lower Confidence Limit; UCL: 95%Upper Confidence Limit; Ref: Reference Category.

The adjusted odds ratios from the multivariate analysis (Table 4) showed that when adjusted for other variables a child had significantly increased odds of being classified as having ADHD if the sex was male (OR 2.82, 95% CI 2.26–3.52); had DEP (OR 5.28, 95% CI 3.65–7.64); had ANX (OR 3.04, 95% CI 2.20–4.19); had INS (OR 1.45, 95% CI 1.00–2.08); watched TV for ≥1 hour (OR 1.32, 95% CI 1.03–1.70); or had someone SMOKE (OR 1.33, 95% CI 1.08–1.64). On the other hand, there were significantly decreased odds of being diagnosed with ADHD if a child was underweight (OR 0.64, 95% CI 0.43–0.95); was either Non-Hispanic Black (OR 0.72, 95% CI 0.53–0.98) or Hispanic (OR 0.65, 95% CI 0.43–0.95); was living in a two-parent FAMILY (OR 0.70, 95% CI 0.56–0.87); or was in SPORTS (OR 0.80, 95% CI 0.65–0.98).

**Table 4:** Adjusted odds ratios for factors associated with ADHD classification, NSCH 2007

Variable	ADHD		
	OR	LCL	UCL
BMI			
Underweight	0.64*	0.43	0.95
Normal	Ref	Ref	Ref
Overweight	1.05	0.81	1.36
Obese	1.06	0.81	1.39
Sex			
Male	2.82*	2.26	3.52
Female	Ref	Ref	Ref
Age	1.00	0.95	1.04
Depression			
Yes	5.28*	3.65	7.64
No	Ref	Ref	Ref
Anxiety			
Yes	3.04*	2.20	4.19
No	Ref	Ref	Ref
Race/Ethnicity			
Non- Hispanic White	Ref	Ref	Ref
Non-Hispanic Black	0.72*	0.53	0.98
Hispanic	0.65*	0.43	0.95
Other	0.78	0.54	1.14
Poverty			
≤200%	1.07	0.84	1.37
>200%	Ref	Ref	Ref
Family members’ smoking status			
At least one member smokes	1.33*	1.08	1.64
No one smokes	Ref	Ref	Ref
Highest level of Education in the household			
Less than/High School Education	Ref	Ref	Ref
More than High School Education	1.11	0.89	1.38
Family structure			
Two parent -biological/step/adopted	0.70*	0.56	0.87
Other - single mother/father/other	Ref	Ref	Ref
Healthcare coverage			
Yes	1.45*	1.00	2.08
No	Ref	Ref	Ref
Participation in Sports			
Yes	0.80*	0.65	0.98
No	Ref	Ref	Ref
Participation in Clubs			
Yes	0.86	0.71	1.04
No	Ref	Ref	Ref
Average computer usage during a weekday			
≥1 hour	1.06	0.85	1.33
<1 hour	Ref	Ref	Ref
Average TV usage during weekday			
≥1 hour	1.32*	1.03	1.70
<1 hour	Ref	Ref	Ref

* Significant association at 0.05 level.

OR: Odds Ratio; LCL: 95% Lower Confidence Limit; UCL: 95%Upper Confidence Limit; Ref: Reference Category.

The multinomial logistic regression model using the trichotomized ADHD classification with medication as the dependent variable showed similar results (Table 5) with some variables such as sex (OR 3.53, 95% CI 2.79–4.46), DEP (OR 6.97, 95% CI 4.66–10.44), ANX (OR 3.38, 95% CI 2.36–4.85), age (OR 0.92, 95% CI 0.86–0.97), INS (OR 2.24, 95% CI 1.24–4.07), and TV (OR 1.56, 95% CI 1.17–2.09) showing significance for the group ADHD-CM, and some variables such as BMI underweight (OR 0.41, 95% CI 0.23–0.75), sex (OR 2.52, 95% CI 1.54–4.14), DEP (OR 4.66, 95% CI 2.57–8.43), ANX (OR 2.77, 95% CI 1.63–4.69), and FAMILY (OR 0.56, 95% CI 0.37–0.85) showing significance for the group ADHD-NCM. In particular, with respect to the variable BMI, obesity was not significant as before while the decreased odds for underweight (OR 0.41, 95% CI 0.23–0.75) was only significant for the ADHD-NCM group. Furthermore, the variables SMOKE and SPORTS lost their significance in this model. Nevertheless, the results from this particular model need to be interpreted with caution due to some limitations to be discussed in the next section.

**Table 5:** Adjusted odds ratios for factors associated with ADHD and Medication use classification, NSCH 2007

Variable	ADHD Not taking Medication			ADHD taking Medication		
	OR	LCL	UCL	OR	LCL	UCL
BMI						
Underweight	0.41*	0.23	0.75	0.82	0.49	1.40
Normal	Ref	Ref	Ref	Ref	Ref	Ref
Overweight	0.84	0.56	1.25	1.00	0.69	1.44
Obese	1.43	0.83	2.47	0.80	0.60	1.06
Sex						
Male	2.52*	1.54	4.14	3.53*	2.79	4.46
Female	Ref	Ref	Ref	Ref	Ref	Ref
Age	1.05	0.96	1.15	0.92*	0.86	0.97
Depression						
Yes	4.66*	2.57	8.43	6.97*	4.66	10.44
No	Ref	Ref	Ref	Ref	Ref	Ref
Anxiety						
Yes	2.77*	1.63	4.69	3.38*	2.36	4.85
No	Ref	Ref	Ref	Ref	Ref	Ref
Race/Ethnicity						
Non- Hispanic White	Ref	Ref	Ref	Ref	Ref	Ref
Non-Hispanic Black	0.78	0.42	1.44	0.74	0.48	1.15
Hispanic	0.90	0.41	1.98	0.61	0.35	1.08
Other	1.00	0.57	1.74	0.70	0.40	1.23
Poverty						
≤200%	1.01	0.66	1.54	1.26	0.91	1.75
>200%	Ref	Ref	Ref	Ref	Ref	Ref
Family Member’s smoking status						
At least one member smokes	1.38	0.99	1.93	1.32	0.98	1.79
No one smokes	Ref	Ref	Ref	Ref	Ref	Ref
Highest level of Education in the household						
Less than/High School Education	Ref	Ref	Ref	Ref	Ref	Ref
More than High School Education	1.03	0.69	1.54	1.13	0.87	1.47
Family structure						
Two parent -biological/step/adopted	0.56*	0.37	0.85	0.89	0.67	1.17
Other - single mother/father/other	Ref	Ref	Ref	Ref	Ref	Ref
Healthcare coverage						
Yes	1.34	0.73	2.44	2.24*	1.24	4.07
No	Ref	Ref	Ref	Ref	Ref	Ref
Participation in Sports						
Yes	0.69	0.47	1.01	0.84	0.65	1.08
No	Ref	Ref	Ref	Ref	Ref	Ref
Participation in Clubs						
Yes	0.83	0.57	1.19	0.93	0.73	1.19
No	Ref	Ref	Ref	Ref	Ref	Ref
Average Computer usage during weekday						
≥1 hour	1.25	0.83	1.87	0.89	0.65	1.22
<1 hour	Ref	Ref	Ref	Ref	Ref	Ref
Average TV usage during weekday						
≥1 hour	1.25	0.76	2.06	1.56*	1.17	2.09
<1 hour	Ref	Ref	Ref	Ref	Ref	Ref

* Significant association at 0.05 level.

OR: Odds Ratio; LCL: 95% Lower Confidence Limit; UCL: 95% Upper Confidence Limit; Ref: Reference Category.

## Discussion

Our comprehensive study found several factors associated with ADHD including some that have not been examined together in conjunction with other variables, especially at the national level. The significant association found between ADHD and DEP, ANX, sex, race, FAMILY, POVERTY, and EDUC is consistent with previous studies on ADHD [22-25]. However, after accounting for the ADHD related factors, obesity was not found to be significant, contrary to some previous studies [25]. The variables SMOKE, INS, SPORTS, and TV were found to be associated with ADHD at the national level for the first time in our study. A child with DEP, ANX, TV ≥1 hour, or with someone SMOKE in household had an increased odds of being diagnosed with ADHD. On the other hand, if a child was underweight, non-Hispanic White, living in a two-parent FAMILY, or in SPORTS, he/she had decreased odds of being diagnosed with ADHD.

This study is not without limitations. The NSCH is a random digital dialing telephone survey based on the responses of parent/guardians. So the responses could be affected by recall bias or the given information could be fallacious (such as misreporting of height/weight). In particular, the diagnosis of ADHD was solely dependent on the response given by a parent to a single question [“Has a doctor or health professional ever told you that S.C. has attention deficit disorder or attention deficit hyperactive disorder, that is, ADD or ADHD?”]; this may have resulted in diagnostic misclassification. In other words, as this is not a clinical study, it is unclear how many children who met the ADHD criteria were undiagnosed and/or untreated. Further, the survey question on SMOKE [Does anyone living in the household use cigarettes, cigar, and pipe tobacco?] does not specify whether the child or someone else in the household including parent/guardian was a smoker; results may alter if the smoker in the household was the child him/herself. Also, some bias is expected due to the cross-sectional nature of the study. For example, the survey fails to capture whether the ADHD, DEP, and ANX diagnosis were concurrent or at different time points in the lifetime of the child. Due to these and the observational nature of the study design, the association found in our study cannot be interpreted as causation for ADHD. For example, the association observed between ADHD and the factors SPORTS and CLUBS could be due to the fact that ADHD diagnosed children are just not welcomed on a sport/club teams because of their behavioral problems rather than lack of sporting/physical activity being a risk factor for ADHD. That is, some of the associated factors could be consequences of having ADHD.

The results show that the ADHD diagnosed children were most likely from a household having insurance. It is not known how many of the children from the uninsured households may have met the ADHD criteria but were undiagnosed. We performed a sensitivity analysis by analyzing only the insured 5–17 years old. The results were similar as before (Table 4) except for minor changes in significance of few variables: TV and race lost their significance marginally while CLUBS gained significance marginally.

With the inclusion of the medication effect, the significance of the association of ADHD with TV and SPORTS was lost. This suggests that these associations could be due to a behavior related factor that could be monitored. However, the results from the model utilizing medication effect may not be totally reliable due to limitations in the medication variable as collected in the NCSH. First, the survey question does not collect information about past medication use for ADHD because of which a child who was diagnosed with ADHD in the past and hence took medication in the past would be categorized into the ADHD-NCM group. While this group is supposed to include only those children who satisfy the conditions of having ADHD and not taking medication for ADHD concurrently. This limitation is similar in essence to the one elucidated earlier due to the cross-sectional nature of the survey. Secondly, the unweighted sample sizes for ADHD-NCM (1,690) and ADHD-CM (3,735) groups do not add up to the total number of ADHD-diagnosed children (7,137 from Table 1) due to missing values for the medication use question. Although our bivariate analysis showed obesity to be significantly associated with ADHD, this was not the case in the multivariate analysis irrespective of whether medication use was considered, contrary to some previous studies [18,21,22,24]. Following Waring and Lapane [24], who had analyzed the NCSH 2003 data, we fitted a model using the same data and with the following subset of variables: sex, race, DEP, ANX, POVERTY, age, and BMI, and the dependent variable as the trichotomized ADHD with medication classification, and indeed found obesity to be significantly associated in this model. However, with the addition of even one or two of the remaining variables, the significance of this association was lost. Thus, our study shows that obesity per se may not have a direct association with ADHD and hence sheds a new light on this research topic.

## Conclusions

ADHD diagnosis and management has been an important feature of child healthcare over the past few decades. Our study uncovered some new factors associated with ADHD at a national level such as TV, SPORTS, SMOKE, and INS after accounting for many other factors. Our findings suggest that children with ADHD are to be monitored for the above factors in addition to the other known factors. This may help pediatricians diagnose and manage ADHD. Further, after accounting for the ADHD related factors, obesity was not found to be significant, contrary to some previous studies. Future research should be directed towards a longitudinal study designed to examine the association between pharmacological factors, ADHD, and related factors in a concurrent manner.

## Abbreviations

ADD: Attention Deficit Disorder; ADHD: Attention Deficit/Hyperactivity Disorder; ADHD-CM: Attention Deficit/Hyperactivity Disorder and currently taking medication; ADHD-NCM: Attention Deficit/Hyperactivity Disorder and not currently taking medication; ANX: Anxiety; BMI: Body Mass Index; CI: Confidence Interval; CLUBS: Participation in Clubs; COMP: Computer usage; DEP: Depression; DHHS: Department of Health and Human Services; DSM: Diagnostic and Statistical Manual of Mental Disorders; EDUC: Education; FAMILY: Family structure; HMOs: Health Maintenance Organizations; INS: Healthcare coverage; POVERTY: Poverty Level; IQR: Inter-Quartile Range; NSCH: National Survey of Children’s Health; OR: Odds Ratio; POVERTY: Department of Health and Human Services Poverty guideline; S.C: Selected Child; SE: Standard Error; SES: Socio-Economic Status; SMOKE: Family member’s smoking status; SPORTS: Participation in Sports; TV: Watching TV/playing Video games.

## Financial Disclosure

The authors do not have any financial interests related to this study.

## Competing interests

None of the authors have any conflict of interest.

## Authors’ contributions

Ravi K. Lingineni reviewed literature, carried out all data analyses, and drafted the manuscript. Swati Biswas supervised Ravi Lingineni in data analysis, interpretation, and critical re-drafting of the manuscript. Naveed Ahmad helped in the conception and design of the study and revision of the manuscript. Bradford E. Jackson helped in data analysis and drafting of the manuscript. Sejong Bae supervised Ravi Lingineni in analysis using complex survey procedures in SAS and revision of the manuscript. Karan P. Singh supervised the whole group, helped in the conception and design of the study, and revision of the manuscript. All authors have approved the final version of the manuscript.

## Pre-publication history

The pre-publication history for this paper can be accessed here:

http://www.biomedcentral.com/1471-2431/12/50/prepub
